# Development of a Combined Protein and Dye Extraction Approach for the Analysis of Keratin-Based Textiles

**DOI:** 10.1021/acs.jproteome.4c00253

**Published:** 2024-08-08

**Authors:** Ilaria Serafini, Gabriele Favero, Roberta Curini, Gwénaëlle M. Kavich, Timothy P. Cleland

**Affiliations:** † Dept of Chemistry, Sapienza University of Rome, Piazzale Aldo Moro 5, Rome 00185, Italy; ‡ Museum Conservation Institute, Smithsonian Institution, 4210 Silver Hill Rd, Suitland 20746, Maryland, United States; § Dept of Environmental Biology, Sapienza University of Rome, Piazzale Aldo Moro 5, Rome 00185, Italy

**Keywords:** keratins, keratin-associated proteins, dyes, archeological textiles, paramagnetic beads

## Abstract

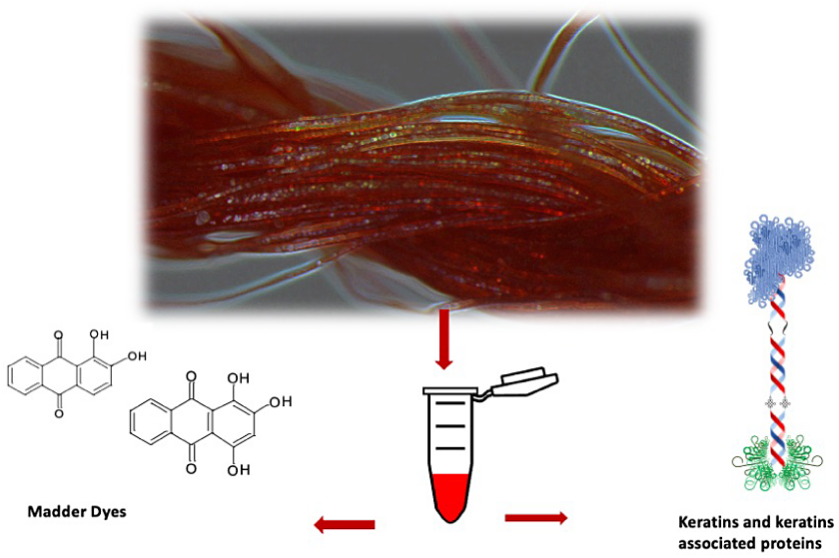

Archaeological textiles represent precious remains from ancient culture; this is because of the historical and cultural importance of the information that can be obtained by such relics. However, the extremely complicated state of preservation of these textiles, which can be charred, partially or totally mineralized, with heavy soil or biological contamination, requires highly specialized and sensitive analytical tools to perform a comprehensive study. Starting from these considerations, the paper presents a combined workflow that provides the extraction of dyes and keratins and keratin-associated proteins in a single step, minimizing sampling while maximizing the amount of information gained. In the first phase, different approaches were tested and two different protocols were found suitable for the purpose of the unique workflow for dyes/keratin-proteins: a slightly modified urea protocol and a recently proposed new TCEP/CAA procedure. In the second step, after the extraction, different methods of cleanup and workflow for proteins and dyes were investigated to develop protocols that did not result in a loss of aliquots of the analytes of interest and to maximize the recovery of both components from the extracting solution. These protocols investigated the application of two types of paramagnetic beads, unmodified and carboxylate-coated hydrophilic magnetic beads, and dialysis and stage-tip protocols. The newly designed protocols have been applied to cochineal, weld, orchil, kermes, and indigo keratin-based dyed samples to evaluate the effectiveness of the protocols on several dye sources. These protocols, based on a single extraction step, show the possibility of investigating dyes and keratins from a unique sample of 1 mg or lesser, with respect to the thresholds of sensitivity and accuracy required in the study of textile artifacts of historical and artistic values.

## Introduction

Mass spectrometric analysis of dyed wool textiles, especially archeological textiles,^1−6^ have, to date, been focused on extracting proteins and dyes separately, consuming larger quantities of materials than a single extraction. This is especially problematic for archeological textiles, usually extremely degraded and subjected to a variety of breakdown processes (e.g., insect damage, fire, carbonization, soil contamination),^2,7−12^ where limited quantities are available for destructive sampling. Hair, the main source of proteinaceous fibers,^10,13,14^ is composed of α-keratins and keratin-associated proteins that have been used for species identification from historical and archeological objects.^1,15,16^ Keratins are a large family of proteins, assembled in coiled-coil heterodimers consisting of one acidic type I subunit (i.e., Ha (K31 to K40)) and one neutral-basic type II subunit (Hb (K81 to K87)). Both subunits range in size from ∼400 to 500 amino acid residues.^13,17^ The heterodimers of intermediate filaments (IFs) associate into tetramers, which connect laterally into an eight-tetramer ring-like monomer, with a final polymerization into keratin intermediate filaments (KIFs).^17,18^ Keratin-associated proteins (KAPs) are a larger group of proteins, divided into 3 main categories: high-sulfur (HSPs), ultrahigh-sulfur (UHSPs), and high glycine-tyrosine (HGTPs) proteins.^13^ The strong association of IFs and KAPs forms hair macrofibrils, whose conformational and mechanical properties are strongly influenced by disulfur bridges and KAP content.^14,17^

In addition to the protein component, textiles are often dyed, and dye identification provides important information about socio-cultural background reconstruction of the textiles.^1,19−25^ Dyes are natural organic compounds, the most common are anthraquinones, flavonoids, carotenoids, indigoids, and phenoxazones and they are of three types based on how they are bound to fibers: direct, vat, and mordant dyes.^19,23^ Direct dyes have sufficient chemical affinity to be bound to the fibers directly. Vat dyes require reduction prior to dyeing the fibers followed by reoxidation and fixation to bind them. Mordant dyes form a complex with a metal cation that then acts as a bridge to the fibers and allows them to fix permanently on them;^23^ they usually represent the most frequent type of dye in textiles. The analytical difficulty in the study of dyes undoubtedly lies in the variety of molecules that contribute to color formation. For example, madder, one of the most common red dyes, has between 45 and 60 species of anthraquinone structures (e.g., purpurin and alizarin) that can affect the dye mixture.^19,26−28^ This therefore determines the work needed on extremely soft and sensitive analytical protocols for enabling them to extract the greatest number of species without altering their composition, which can give useful information on the origin of the matrix used.^22,29−32^

In the last few years, research in the field of proteomics and the study of dyes has progressed on parallel tracks, developing in both cases extremely sensitive to reduce invasiveness and especially sample size, taking into account the fact that these are extremely valuable, artistic, and historical relics, from which it is generally not possible to have more than a few milligrams of samples. In this perspective, the lack of an investigation protocol or approach that considers both components, dyes and proteins, with the aim of performing comprehensive characterization from a single sample.

Starting from these considerations, this study focuses on the development of a protocol that can perform the extraction of both components of wool textiles, dyes, specifically anthraquinone ones (e.g., madder, cochineal) and keratins and keratin-associated proteins, evaluating how a common protocol might affect the yields of the two components. The method, initially developed on the most common anthraquinone dyes (madder), is then applied to cochineal, weld, kermes, indigo and orchil to evaluate its effectiveness on different dye sources. Furthermore, throughout the development process, various protocols and combinations of cleanup methods for dyes and proteins are assessed. This included the exploration of protocols typically unused for keratins, such as paramagnetic nonfunctionalized beads, as well as the examination of μSPE or stage-tip protocols for dyes. At the end, we demonstrate that it is possible to achieve extraction and characterization, with results on par with those of individual analytical processes, in some cases dramatically cutting down sample processing times and especially the number of samples required.

This paper represents the first phase of development of the PARCA project, funded by the European Commission (grant agreement no. 101029204), which aims to first work from the standpoint of method development on dyes and proteins and then, once the protocol has been identified, to study and characterize the proteomics and dyes from charred textile artifacts from some areas in the north Mediterranean.

## Experimental Procedures

### Undyed and Dyed Wool Samples

Experiments were performed on undyed and dyed commercial wool (sheep) samples, prepared in lab.

Dyeing process: the dyeing process with madder roots, cochineal insects, indigo powder, weld areal parts (purchased from Kremer Pigmente), and orchil lichens following the protocol described in the literature,^23,33^ with the mordanting process and dyeing bath, separately.

Mordanting process: first, the mordanting bath was prepared with 31% alum (KAl(SO_4_)_2_) in relation to the weight of dry wool (1 g) and 6% cream of tartar (C_4_H_5_KO_6_) in 250 mL of deionized water. This solution was warmed at 40 °C for 10 min to achieve complete solubilization of the salts. Then, it was maintained slightly cool, and the soaked wool was added. The temperature was gradually raised to 80 °C, and the wool was maintained in the bath at this temperature for 1 h under gentle magnetic stirring. After 1 h, the bath was cooled to room temperature before removing the wool, and then, the excess moisture was squeezed out and the wool was left to dry.

Dyeing process: a dyeing bath (400 mL) was obtained with dried madder roots (madder roots:wool, 1:1 ratio, w/w) in water or with dried cochineal insects (insects: wool, 1:1 ratio, w/w) dipped in water. The soaked wool (1 g) was introduced into a lukewarm bath, and the mixture was heated to 80 °C in 40 min. This temperature was maintained for 1 h under gentle magnetic stirring. After that, the wool was left to cool in the bath for 30 min and then removed and rinsed many times until the washing water appeared completely colorless. The yarns were then left to dry.^23,33^

For weld-dyed wool, a first dyeing bath (400 mL) was obtained by adding 5g of aerial parts of *Reseda luteola* L., dried and powdered, and then dipped in distillated water. The dye bath was heated to 80 °C in 40 min, and this temperature was maintained for 1 h under gentle stirring. Then, the solution was left to cool and filtered. A second dye bath was prepared in the same way. The soaked wool (1g) was introduced into the first dye bath and left for 12 h. Then, the wool was dipped in the second dye bath for other 12 h. In both cases, the dye bath was maintained at room temperature. After that, the wool was removed and rinsed many times, until the washing water appeared completely colorless. The yarns were then left to dry.^23^

To dye with indigo, 0.6 g of pigment ground into a very fine powder was used and added to 10 mL of distilled water previously heated to 45 °C. After that, to the indigo solution were added an aqueous solution containing 0.6 g of sodium carbonate in 6 mL of distilled water, 1.5 g of sodium dithionite and 50 mL of water, all of them preheated to 40–50 °C. This was brought to 55 °C and left at temperature for 20 min, after which 3 g of wool was immersed in the color bath and left to soak for 10 min. The wool was then taken out and allowed to air dry, so that the dye could oxidize again and achieve the final color. Finally, the yarn was rinsed with distilled water until it was clear and allowed to dry.^23^ For orchil-dyed wool, nonmordant wool and mordant wool were dyed. Since the lichens do not contain dye molecules but a precursor, a pretreatment of the lichens is necessary to obtain the color and the dye bath was prepared as follows: 25 g of areal parts of the lichens were dipped in a solution of 300 mL of 30% NH_3_ and 1.5 mL of 0.73 mg/mL K_2_CO_3_. The lichens were maintained in this solution in the dark for 20 days under mechanical stirring two or three times every day.^23^

After this time, the lichens were removed, and the solution was air-flowed to remove NH_3_. Then, the mordant wool was dipped in the proper dying bath, prepared with 100 mL of stock solution and diluted to 300 mL with deionized water. The solution was brought to the boiling temperature. The color was controlled every 15 min, until reaching the desired color (dark purple), and generally, the process took 45 min. After that, the solution was left to cool down; then, the wool was removed and rinsed many times until the washing water appeared completely colorless. The yarns were then left to dry overnight.^23^

### Reagents

Urea, Trizma (TrisHCl), and iodoacetamide (IAM) were obtained from Sigma-Aldrich. Tris(2-carboxyethyl)phosphine hydrochloride (TCEP HCl) was obtained from Thermo Scientific, while 2-chloroacetamide (CAA), 98+% from MP Biomedicals LLC. Formic acid (FA), optima LC-MS water, acetonitrile (ACN), methanol (MeOH), ammonia (NH_3_ 30%), and sodium EDTA were obtained from Fisher Chemical. Ammonium bicarbonate (AmBic) was obtained from VWR International LLC. Ethanol (EtOH) was obtained from EMD Millipore Corporation (Burlington, MA). MINI Dialysis units Slide-A-Lyzer were purchased from Thermo Scientific. Empore C18 SPE Extraction disk and Empore SDB-RPS Extraction disk were purchased from 3 M Bioanalytical Technologies (St. Paul, MN). For the paramagnetic beads, Sera-Mag Speedbeads were purchased from GE Healthcare and SeraSil-Mag 700 from Cytoviva.

### Extraction Protocols

#### Simultaneous Extraction of Proteins and Dyes

To evaluate the best conditions for the simultaneous extraction of dyes and proteins, different protocols have been investigated.

##### Urea Extraction Protocol

1 mg of yarns (both dyed and undyed yarns were subjected to the protocol, separately) were extracted for dyes and proteins in 100 μL of 8 M urea, 50 mM Tris hydrochloride, 50 mM TCEP·HCl, and 1 mM Na_2_EDTA. The pH was checked and a few microliters of NaOH were added to achieve the pH value of 8–9. The solution was left to shake constantly overnight (≥18 h). After this time, 25 μL of the same buffer were added to the samples for another 2 h of extraction on the shaker. After centrifugation at 2800 rpm, 100 μL of the supernatant was collected and used for the alkylation and eventually dye cleanup.^15^ The solution was alkylated for 45 min in the dark with 10 μL of 400 mM iodoacetamide under shaking. After alkylation for dye analysis, the solution was subjected to the dye purification through μ-SPE, dLLME, or stage-tip protocol, leaving a dye filtered solution that was retained for protein in-solution digestion. Also paramagnetic beads, single-pot solid-phase-enhanced sample preparation (SP3)^34−37^ and Bead-enabled Accelerated Monophasic Multiomics (BAMM) protocol,^38^ were employed, and in this case, the proteins were loaded on the beads and then directly subjected to on-bead digestion. The remaining solution, dyed, was subjected to dye purification.

When the dye was filtered, aqueous solution was retained for protein digestion, and the solution was desalted using Slide-A-Lyzer dialysis (3.5 K MWCO) cassettes against 100 mM ammonium bicarbonate at pH 8.0 for 2 h, followed by overnight dialysis in a new ammonium bicarbonate solution. The dialyzed solution was then digested overnight with 0.5 μg of trypsin at 37 °C. After about 16 h, the samples were acidified with 10 μL of 1% FA and the proteins were selectively recovered by solid phase extraction.

For single-pot solid-phase sample preparation (SP3)^35^ with Sera-Mag beads or the BAMM method with SeraSil-Mag 700 beads,^38^ the subsequent protocols have been followed. For SP3,^34−37^ a 40 μL mixture of hydrophilic and hydrophobic Sera-Mag Speedbeads (1:1) was washed three times in water. To recover the proteins from the solution, 10 μL of the prepared beads were added to the solution along with 120 μL of 100% ethanol and incubated for 10 min at room temperature. After incubation, beads were placed on the magnetic rack for 2 min to collect the beads, and the supernatant was removed and discarded. The beads were subsequently washed three times with 200 μL of 80% ethanol and incubated on the magnetic rack for 2 min, and the supernatant was discarded.^34,37^ At the end, the samples were left to dry for a few minutes before digestion. 95–100 μL of 50 mM ammonium bicarbonate solution were added to the samples, together with 0.5 μg of trypsin, for overnight digestion at 37 °C. The digestion was then stopped with 1% FA, and the supernatant was collected after incubation for 2 min on the magnetic rack. The samples were then processed for purification with solid-phase extraction^15^ or the stage-tip protocol.

For BAMM,^38^ 30 μL of SeraSil-Mag 700 were washed twice with Milli-Q water and stored until use. After alkylation with iodoacetamide, 10 μL of SeraSil-Mag beads were added to 90 μL of the extracting solution, together with 100 μL of ACN and 300 μL of BuOH, following the proportion for the monophasic extraction with beads, where the ratio of *n*-butanol/ACN/H_2_O should be 3:1:1.^38^ In this case, the dye purification has been evaluated before and after the beads’ purification (After beads’ purification, the supernatant was collected and dried down for 1 h at 45 °C under vacuum, and then resuspended and subjected to μSPE, the stage-tip protocol, or dLLME).

The beads were washed once with 100 μL of water and then left to dry. Once the solution was dried, 95 μL of 100 mM ammonium bicarbonate solution and 0.5 μg of trypsin were added for overnight digestion. After 18 h, the digestion was stopped with 10 μL of 1% FA and peptides were purified with the stage-tip protocol or solid phase purification.

For insoluble fibers remaining after extraction, the pellet was alkylated in 50 μL of 40 mM iodoacetamide for 45 min in the dark. After this, 100 μL of 100 mM ammonium bicarbonate were added and digested directly with 0.5 μg of trypsin.^15^

##### TCEP/CAA

100 μL of 100 mM TCEP/400 mM CAA were added to the samples, incubated on a thermomixer for 10 min at 95 °C.^15^ After that, proteins were separated from the solution through SP3 (as described above) or directly subjected to trypsin digestion.

### Protein Purification after Digestion

#### Solid Phase Peptide Purification

Peptide purification solid phase extraction^15^ was conducted with the membrane of Empore C18 SPE Extraction Disk; disks of 0.1 cm were cut, then washed with ACN (1 min), conditioned with MeOH (1 min), and washed again with 0.1% FA solution (1 min). For each sample, a disk was added to the solution and left for 3 h with light shaking to allow protein loading on the disk. Following a brief wash of the disk in a new tube with 100 μL of 0.1% FA solution (1 min), the peptide mixture was then eluted by leaving the disk in 100 μL of 75:25 (v/v) ACN:0.1% FA for 1 h. All samples were then dried on a Speedvac vacuum concentrator and resuspended in 10 μL of 0.1% FA.

#### Stage Tipping Protocol

The stage-tip protocol was used in some cases as an alternative to the solid-phase purification . It was used with the octyldecylsilane (C18) phase for peptide purification, while both C18 and polystyrene-divinylbenzene reversed-phase sulfonate (SDB-RP) were also evaluated in the dye purification and preconcentration step (before digestion).^34,39^ Three Empore C18 disks were cut and placed all stacked together in 200 μL of tips. The tip was then placed in the lid of a 2 mL tube. Tips were first washed with 20 μL of 100% methanol and then 20 μL of 80% acetonitrile with 0.1% formic acid and finally equilibrated with 20 μL of 0.1% formic acid. Samples were added and then washed with 0.1% formic acid. When the stage tip was used to purify peptides, all peptides were eluted with 80% acetonitrile with 0.1% formic acid and then dried under vacuum. The final peptide samples were resuspended with 10 μL of 0.1% formic acid and injected in a LC-MS. When stage tips were used to purify and concentrate dyes, the elution solvent was a mixture of MeOH:H_2_O:FA, 80:15:5,^40^ and the solution was directly injected in the LC-MS.

### Dye Cleanup

#### Liquid–liquid Extraction: LLE

To separate the dye, the solution was acidified to pH 3 with 2 N HCl. Then, a liquid–liquid extraction was performed by adding 50 μL of 1-pentanol, shaking, and then waiting for the separation of the phase. This step was repeated twice. The two fractions of 1-pentanol were reconstituted together, dried and resuspended in 40 μL of MeOH:H_2_O (1:1), and injected into the column.

#### Microsolid-Phase Extraction (μ-SPE)

The cleanup of the anthraquinone dyes was performed through the Pierce C18 tips. The different steps were performed as follows: the functionalized fiber was activated by flushing 100 μL of MeOH for 3 cycles (loading and unloading). The conditioning step was achieved by flushing a solution of 8 M urea, 50 mM Tris hydrochloride, 50 mM TCEP·HCl, and 1 mM Na_2_EDTA for 3 cycles; then, the samples were loaded flushing the solution for 5 cycles. After a brief wash of the tip with 0.1% FA, the elution was performed with 40 μL of MeOH:H_2_O:FA, 80:15:5,^40^ for 5 cycles. Once the elution was completed, the tip was discarded, and the solution obtained was directly injected into the column.

### dLLME: Dispersive Liquid–Liquid Microextraction

The cleanup followed these steps: the extracting solution was brought to the volume of 1.6 mL, and 500 mg of NaCl, 1 mL of 6 M HCl, and 0.8 mL of formic acid were added to the extraction solution, alongside 250 μL of 2-propanol. 200 μL of 1-pentanol and 100 μL of 2-propanol were then rapidly injected to obtain a cloudy solution. The samples were vortexed before being placed in an ultrasonic bath for 10 min. After sonication, the mixture was centrifuged at 12 500 rpm for 10 min to separate the layers. The aqueous layer was removed, and 1-pentanol was washed using NaCl in Millipore water (166 mg/mL) until a pH value of 4.5–5 was achieved. The organic phase was then transferred into a vial and dried by heating at 65 °C. The extract was reconstituted with 100 μL of H_2_O:methanol (1:1) for analysis by HPLC-MS.^41,42^

### Sequential Extraction of Dyes and Proteins

#### Ammonia Extraction for Dyes and TCEP/CAA

To extract the dyes, prior to protein extraction, the dyed yarns were dipped in a solution of 30% NH_3_ solution and 1 mM Na_2_EDTA solution for 48 h at room temperature, following the general ratio 1 mg of sample, 0.8 mL of NH_3_, and 0.8 mL of 1 mM disodium EDTA + 4.4 mg NaCl.^21^ After that time, the solution was collected and subjected to dLLME.^41,42^ To the extracting solution, 495.6 mg of NaCl was added together with 1 mL of 6 M HCl and 0.8 mL of HCOOH (≥95%) to bring the solution pH to 3. The dyes were then extracted from the aqueous phase into the organic solvent: 250 μL of 2-propanol was added to every sample and, subsequently, together in the same syringe, 200 μL of 1-pentanol and 100 μL of 2-propanol were vigorously injected to obtain a highly dispersed three-phasic system, known as a cloudy solution.^43^ After the ammonia extraction for dyes, the yarns were washed with Milli-Q water to remove the residual ammonia and salts, and then dried and subjected to TCEP/CAA and SP3, digestion, and protein purification as described above.

#### Second Ammonia Protocol from Dyes and Proteins

The ammonia method proposed by Andreotti et al.,^44^ was initially considered for protein binders’ identification and here applied to evaluate its effectiveness in extracting keratin proteins. The protocol was applied as described: first, on 1 mg of samples, the ammonia extraction protocol was applied as described above. The aqueous solution was removed, and dLLME was performed to recover the dyes. The yarn was then incubated in 200 μL of 2.5 M NH_3_; then, the solution was maintained at 60 °C for 120 min. The process was repeated twice. The two solutions obtained were reconstituted together and dried, and then resuspended in 100 μL of 100 mM TCEP/400 mM CAA at 95 °C for 10 min to achieve the reduction and alkylation of proteins. After this, SP3, digestion, and solid phase purification were applied.

##### LC-MS/MS

Peptides (1 μL) were separated on ThermoScientific Acclaim PepMap 100 trap columns (100 μm i.d. × 2 cm, 5 μm particle size) and separated on a ThermoScientific Acclaim PepMap RSLC analytical column (75 μm i.d. × 25 cm, 2 μm particle size) at 300 nL/min using a ThermoScientific Dionex Ultimate 3000 UHPLC system (2% B 0–8 min, 55% B 98 min, 90% B 100–103 min, 2% B mount permeation 104–120 min, buffer A-0.1% FA in H_2_O; buffer B-0.1% FA in ACN). Dyes were separated with a Waters BEH Shield RP18 at 0.2 mL/min (buffer A: 0.1% FA in H_2_O; buffer B: 0.1% FA in ACN using a gradient elution according to the following steps: 10% B 0–3 min, 16% B 4–7 min, 35% B 7–12 min, 60% B 12–17 min, 85% B 17–28 min, 99% B 29–33 min, 10% B 35–40 min). For both dyes and proteins, the UHPLC was directly coupled to a Thermo Scientific Orbitrap Elite mass spectrometer with the parameters listed in Table 1.

**Table 1:** MS Parameters for Dye and Proteins Analyses

	ion source	mode	MS1 resolution	max inject time(ms)	automatic gain control(AGC)	MS2 resolution	max inject time (ms)	automatic gain control	TOP	normalized collision energy (NCE)(HCD)	collision energy (CID)
dyes	HESI	-	60	0.5	1 × 10^6^	15	100	5 × 10^4^	3/3	35	35
proteins	nESI	+	60	100	1 × 10^6^	15	250	5 × 10^5^	10	35	-

##### PEAKS X: Protein Identification Software Tool

PEAKS X Pro (Bioinformatics Solutions Inc.) was used to search the RAW data against an *Ovis aries* keratin and KAP database (downloaded August 2021). Searches were carried out using trypsin as the enzyme; one allowed nontrypsin cleavage at any end and two missed cleavages with a precursor mass tolerance of 10 ppm and a fragment mass tolerance of 0.02 Da. Carbamidomethylation was chosen as a fixed modification for all samples, and deamidation (NQ), oxidation (M), pyroglu (Q), and carbamylation as variable modifications. A maximum of three PTMs were allowed. The protein score threshold was set at −10lgP = 20 and peptide score at −10lgP = 15, with a minimum of 2 peptides. The mass spectrometry proteomics data have been deposited on MassIVE, available under MSV000093518 and MSV000095268.

Evaluation of the levels of deamidation was calculated as the percentage of the total number of deamidated asparagine (N) and glutamine (Q) residues to the total of N and Q residues in the peptides identified.^15^

##### Qualbrowser: Dye Identification

Dyes were analyzed through Qualbrowser, and their identification was based on the MS/MS database and literature (Table 2) or through Skyline version 20.2.

**Table 2:** List of Some of the Most Common Madder Dyes Compounds; Their Elemental Formula, Molecular Weight, and Fragmentations Were Available in the Literature.^26,30,31,32,45,46,47^

compound name	elemental formula	monoisotopic mass (g/mol)	mass fragmentation
Madder dye compounds			
alizarin (quinizarin, xanthopurpurin)	C_14_H_8_O_4_	240.04225873	211, 210 [45]
purpurin (anthragallol)	C_14_H_8_O_5_	256.03717335	227, 171, 129, 101 [45]
lucidin (anthragallol methyl ether)	C_15_H_10_O_5_	270.05282342	251, 223, 195 [45]
munjistin (christofin, lucidin ethyl ether)	C_15_H_8_O_6_	284.03208797	299.1; 255.2; 226.9; 158.8 [26]
rubiadin	C_15_H_10_O_4_	254.05790880	225, 209, 195 [45]
chrysazin	C_14_H_8_O_4_	240.04225873	211 [45]
munjistin methyl ester	C_16_H_10_O_6_	298.04773803	
alizarin glucoside	C_20_H_18_O_9_	402.09508215	239
purpurin glucoside	C_20_H_19_O_10_	419.09782180	255
lucidin glucoside	C_21_H_20_O_10_	432.10564683	269
ruberythric acid	C_25_H_26_O13	534.13734088	239 [45]
lucidin O-primeveroside	C_26_H_28_O_14_	564.14790556	269; 251 [45]
rubiadin O-primeveroside	C_26_H_28_O_13_	548.15299094	253 [45]
Weld compounds			
apigenin	C_15_H_10_O_5_	270.0528234	117 [46]
luteolin	C_15_H_10_O_6_	286.047738	133 [46]
chrysoeriol (diosmetin)	C_16_H_12_O_6_	300.0633881	284 [46]
quercetin	C_15_H_10_O_7_	302.0426527	179, 151, 107 [46]
apigenin monoglycosylated	C_21_H_20_O_10_	432.1056468	269, 268 [46]
luteolin monoglycosylated	C_21_H_20_O_11_	448.1005615	285 [46]
chrysoeriol monoglycosylated	C_22_H_22_O_11_	462.1162115	285
quercetin monoglycosylated	C_21_H_20_O_12_	464.0954761	301, 300, 271, 243 [46]
apigenin biglycosylated	C_27_H_30_O_15_	594.1584703	285
luteolin biglycosylated	C_27_H_30_O_16_	610.1533849	284, 255
quercetin biglycosylated	C_27_H_30_O_17_	626.1482995	
Cochineal dye compounds			
dc1	C_22_H_19_O_12_	476.0995	431, 311, 282 [32, 47]
dc2	C_22_H_18_O_14_	506.0697	477, 433, 343 [32, 47]
dcII	C_22_H_20_O_12_	476.0955	431, 341, 311, 282 [32, 47]
ca	C_22_H_20_O_13_	492.0904	447, 357, 327 [32, 47]
dcIII	C_22_H_20_NO_12_	491.1064	446, 356, 326 [32, 47]
dc3	C_22_H_24_O_14_	536.1166	473, 445, 415 [32, 47]
dcofk	C_22_H_20_O_12_	476.0955	431, 268 [32, 47]
dc4	C_23_H_20_O_14_	520.0853	397, 385, 327 [32, 47]
dc5	C_22_H_18_O_13_	490.0741	487, 399, 369 [32, 47]
dcIV	C_22_H_20_O_13_	492.0904	447, 357, 327, 284 [32, 47]
dc6	C_23_H_20_O_14_	520.0853	487, 399 [32, 47]
dc7	C_29_H_23_O_15_	612.1115	429, 309 [32, 47]
dc8	C_21_H_20_O_11_	448.1006	447, 357, 327 [32, 47]
dcVII	C_22_H_20_O_13_	492.0904	357, 327, 299 [32, 47]
dc9	C_29_H_23_O_15_	612.1115	567, 429, 257, 327 [32, 47]
fa	C_16_H_10_O_7_	314.0427	269, 257 [32, 47]
ka	C_16_H_10_O_8_	330.0376	285, 257 [32, 47]
pp1	C_28_H_30_O_18_	654.1432	
pp2	C_27_H_30_O_15_	594.1585	473, 431, 269 [32, 47]
pp3	C_28_H_30_O_18_	654.1432	609, 357, 327, 299 [32]
pp4	C_28_H_30_O_18_	654.1432	
pp5	C_28_H_30_O_18_	654.1432	620, 609, 533, 491, 473, 447 [32, 47]
pp6 (ppI)	C_22_H_20_O_12_	476.0955	431, 269 [32, 47]
pp7 (ppII)	C_22_H_20_O_13_	492.0904	447, 285 [32, 47]
pp8	C_22_H_20_O_12_	476.0955	431, 268 [32, 47]
pp9	C_22_H_20_O_13_	492.0904	447, 284 [32, 47]
pp10	C_21_H_20_O_10_	432.1056	431, 269 [32, 47]
pp11	C_24_H_22_O_14_	534.101	489, 357, 327, 299 [32, 47]
pp12	C_21_H_20_O_10_	432.1056	268, 240 [32, 47]
pp13	C_21_H_20_O_10_	432.1056	268 [32, 47]
pp14	C_28_H_30_O_14_	590.1636	545, 357, 327, 299 [32, 47]
doe	C_15_H_10_O_5_	270.0528	
Indigo dye compounds			
isatin	C_8_H_5_NO_2_	147.032028402	130, 102, 92, 77, 65 [45]
indigotin	C_16_H_10_N_2_O_2_	262.074227566	235, 219, 206, 132, 77 [45]
indirubin	C_16_H_10_N_2_O_2_	262.074227566	235, 219, 206, 190 [45]
Orcein dye compounds			
α-amino orcein	C_21_H_18_N_2_O_4_	362.12665706	348, 240 [30–31]
α-hydroxy orcein	C_21_H_17_NO_5_	363.11067264	349, 241 [30–31]
β/γ amino-orceinimine	C_28_H_25_N_3_O_5_	483.17942091	468 [30–31]
β/γ hydroxy orcein	C_28_H_23_NO_7_	485.14745207	470, 454, 4245, 362, 346, 333 [30–31]
β/γ amino-orcein	C_28_H_24_N_2_O_6_	484.16343649	469, 453, 424, 361, 345, 332 [30–31, 45]

## Results and Discussion

Different protocols were studied in the initial stage of the work to find the best protocol to achieve the simultaneous extraction of proteins and dyes from madder dyed wool. At the end, two different protocols seemed to give the best performances, the new TCEP/CAA method at 95 °C for 10 min^15^ or a modified urea method. Once identified the efficient extracting solutions, several digestion methods were investigated: proteins were digested with trypsin either in-solution after dialysis or with single-pot solid-phase-enhanced sample preparation (SP3)^34−37^ or through the application of unmodified paramagnetic beads on keratin proteins.^38^ Then, the solution was desalted with C18 SPE or C18 stage tips. Dyes were desalted, preconcentrated and purified, evaluating various cleanup methods, such as liquid–liquid extraction, μ-SPE, dispersive liquid–liquid microextraction (dLLME)^41,42^ or stage tips with different membranes.

As described above, the importance of developing a single protocol for the simultaneous analysis of textile fibers from a proteomic and dye perspective lies in several areas. Given the complexity of the artifacts from a chemical point of view, with the complexity of the diagenetic processes to which they are subjected during their “life” until their excavation, any information is definitely valuable in reconstructing their history. This ambitious goal goes through the characterization of all components. Moreover, because of the preciousness of the material, it is not always possible to obtain multiple samples for proteomic and dye analyses. In this paper, the protocols presented enable minimization of the amount of samples needed while obtaining the maximum amount of information possible.

**Figure 1:**
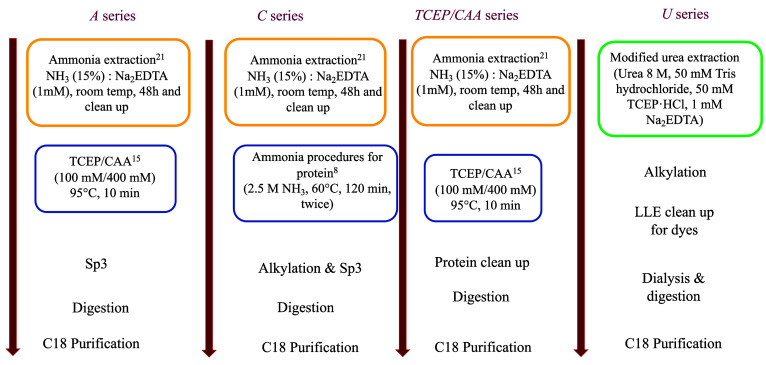
Scheme of the first tests carried out to identify the best extraction protocol. Blue circles highlight the protein extraction method; the yellow circles, the dye extraction; the green one, the single extraction.

**Figure 2:**
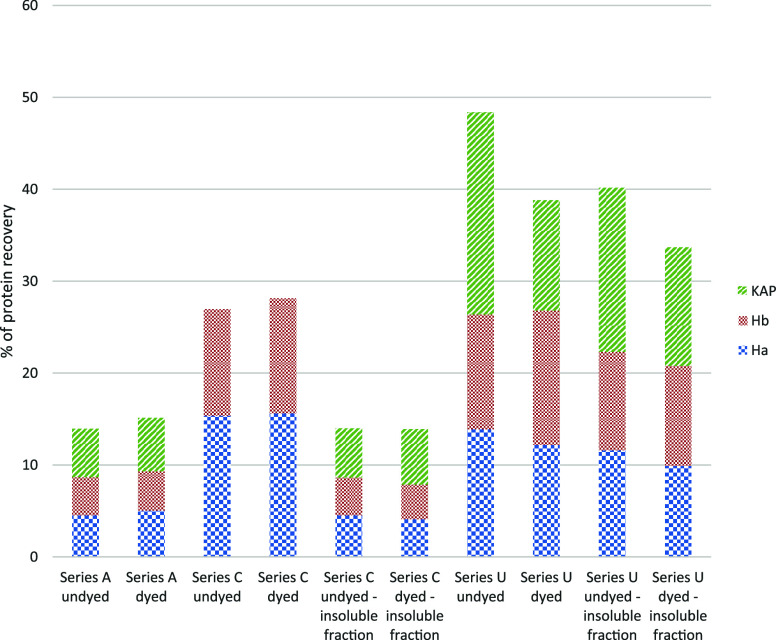
Percentage of protein recovery for the first two trials.

**Figure 3:**
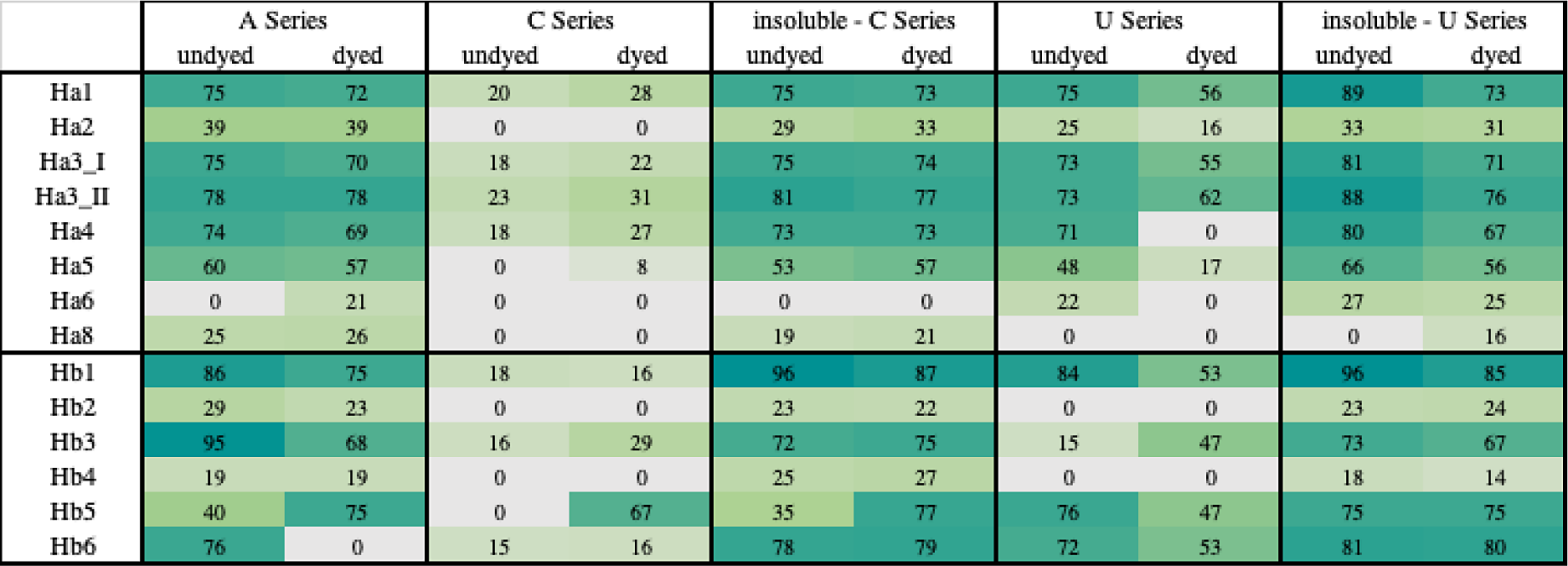
Heath map of coverage % for acid and basic keratins.

In the first phase of the research of trials, two different approaches have been considered. The first approach is based on the application of a protocol specifically designed for dye extraction that was first applied and then in series the application of an extraction protocol for wool proteins. The dye protocol using 15% NH_3_ with Na_2_EDTA^21,33^ was chosen for the following advantages: the method does not involve a hot extraction process, which could induce further modification, and it is proved to be particularly mild toward the dye species, preserving as much as possible their molecular pattern fixed on the fiber, including the most fragile glycosylated components.^21^ After this, the samples were subjected to the novel TCEP/CAA extraction method,^15^ which applies a unique step for reduction and alkylation (as extraction step) at 95 °C with a higher concentration (100 mM and 400 mM for TCEP and CAA, respectively). After this, SP3 purification was applied, followed by trypsin digestion and C18 peptide purification. This series is called series A (Figure 1); for each series, two samples, dyed and undyed ones, were always considered to evaluate the performances of the method in both cases with an undyed sample as reference (Figures 2 and 3).

In the same type of approach, there is a second set of tests that instead aimed to apply an ammonia methodology previously used for protein binders and evaluate its effectiveness for keratins and keratin-associated proteins.^44^ Similar to series A, the ammonia method for dyes was first applied, and then the ammonia method for proteins was carried out. If the method showed promising results in extracting the protein component of wool, relying on the same type of extractant as the ammonia method, it might have been thought to join the two protocols to achieve a common one effective on both components. This is series C; the analytical steps are shown in Figure 1, and the results are shown in Figures 2 and 3.

The second approach was directly focused on developing conditions suitable for the extraction of both components simultaneously. The ammonia method suggested that an alkaline environment could be effective for extracting anthraquinone dyes or dyes, in general, with hydroxyl substituents. For this reason, the urea extraction method provides an alkaline environment at around pH 8–9,^48−50^ seemed to offer a sufficiently basic environment for extraction. Na_2_EDTA was added to break down the metal complex that keeps the mordant dye attached to the fiber. Once the extraction with urea-Na_2_EDTA was performed, the solution was alkylated with iodoacetamide. After alkylation, the solution was red in color and was subjected to LLE to isolate the dyes. Once the dyes were separated to the organic phase and removed, the proteinaceous aqueous phase was dialyzed to remove also HCl used for LLE and then digested with trypsin and peptides purified with C18 solid purification. This is series U.

The different tests are summarized in Figure 1, while the results are presented in Figures 2 and 3.

For the A series, the association of the ammonia protocol for dyes with TCEP/CAA seems to be effective in extracting dyes and protein, being able to characterize several Type I (including minor components Ha2, Ha6, and Ha8) and Type II (including Hb2, Hb4) proteins, and KAPs. However, the method showed two major limitations: first, the application of the ammonia method for dyes requires that the yarn must be rinsed before undergoing the next extractive step in TCEP/CAA. This washing might not be feasible in the case of an extremely degraded archeological textile artifact. In addition, the deamidation value for these samples is higher than expected, by about 16–20%, which is the deamidation value expected from archeological textiles.^15,50,51^ This could be related to the presence of ammonia that can increase the rate of deamidation enhanced by high temperature with ammonia,^52^ which might be not completely removed by just washing.

The C series seems to give poor results in extracting keratin proteins. Few Ha and Hb were detected, and there was no KAP. Furthermore, even those that were extracted showed lower levels of sequence coverage. Also, the insoluble fractions were analyzed with definitely better results, and it is clear that the highest number of proteins were not been extracted by the ammonia protocol for binders, but were successfully recovered only by further extraction of the insoluble fraction. The values of deamidation were higher than 20%, as expected for the ammonia treatment at a high temperature.

The urea method with the addition of Na_2_EDTA seems to be very promising in creating a unique workflow. In the first attempt, the fiber was left in the urea solution for 48 h as for dyes extracted in ammonia, but it was already colored after 18 h, so a two-day extraction is not needed. This means that this methodology in a basic environment is faster than ammonia for extraction of dyes. Nonetheless, even if the solution appeared strongly colored, the cleanup method for dyes caused some issues: LLE (so basically slightly acidifying the solution and then adding 1-pentanol-organic phase) was initially considered for higher volumes, but such small quantities were very difficult to handle. Thus, in removing pentanol, some aqueous aliquots were removed, and some peptides were probably lost. This explains why some Ha and Hb seem to be missing, such as the minor components Ha 4, 6, and 8 and Hb2 or 4. Insoluble fractions in this case did not give better results, showing a general lower number of keratins and KAPs (Figure 1).

It should be noted that dyed samples showed lower protein recovery compared to undyed samples, suggesting that dyeing processes induce some processes that change the protein structures (deamidation values are always higher for dyed samples) and reduce the accessibility of proteins in the extraction process.

Following the initial investigations, both ammonia-based methods (series A and C) were abandoned. The final protocols were designed to perform well against dyes and keratins. Specifically, focus was placed on the urea–Na_2_EDTA and just TCEP/CAA. We tested these protocols for their ability to simultaneously extract proteins and dyes. For TCEP/CAA, it was taken into account that the acid conditions at high temperature could still induce dye extraction. Many extraction methods for dyes use indeed the acidic environment at high temperatures.^53−58^ It should be noted that generally even soft acid treatment can lead to dye alteration, especially in glycosidic bond breaking. However, some considerations can be made: first, Solazzo et al.^15^ showed that the urea method, which is generally very effective on textile artifacts, is inefficient on textile artifacts that are heavily affected by soil contamination. In these cases, the TCEP/CAA method resulted in a higher recovery, suggesting that the heating step increased denaturation of the proteins, making them more accessible for protein digestion, despite the soil contamination.^15^ In this sense, the possibility of having two protocols equally or with similar yields can represent a versatile tool that can be used in one way or another depending on the state of preservation of the textile artifact. Furthermore, despite acidic methods inducing changes to the molecular pattern of dyes, it is likely that in certain types of preservation (e.g., charred), the few dye molecules are mostly aglycone and therefore less subject to acidic extraction-induced changes.

This is why evaluating the possibility of analyzing and extracting dyes, even when present in traces and perhaps barely visible to macroscopic observation, is an important step in the investigation of ancient archeological textiles. In fact, no study to date has evaluated or developed a method that would allow the two components to be analyzed with the ultimate goal of not losing analytical sensitivity for both. For this reason, for both the urea and TCEP/CAA extraction protocols, different combinations of extraction and purification protocols were investigated, to ensure high analytical sensitivity, and compared with a reference sample (in this case without any evaluation of dye recovery, just focusing on keratin and KAP identification). For the urea protocol, both dialysis and SP3 were considered for protein purification. For TCEP/CAA the lowest and highest concentrations (10 mM/40 mM and 100 mM/400 mM) were evaluated with or without SP3, similarly to Solazzo et al.^15^

The same considerations were made for dye isolation; since LLE was ineffective, two approaches were evaluated: loading the proteins onto the paramagnetic beads and leaving the dyes in the aqueous solution or selectively recovering the dyes by μ-SPE or stage tips with either C18 or SDB-RPS (polystyrene-divinylbenzene, reversed-phase sulfonate) membranes, thus separating the proteins from the dyes allowing for the normal in-solution digestion protocol.

**Figure 4:**
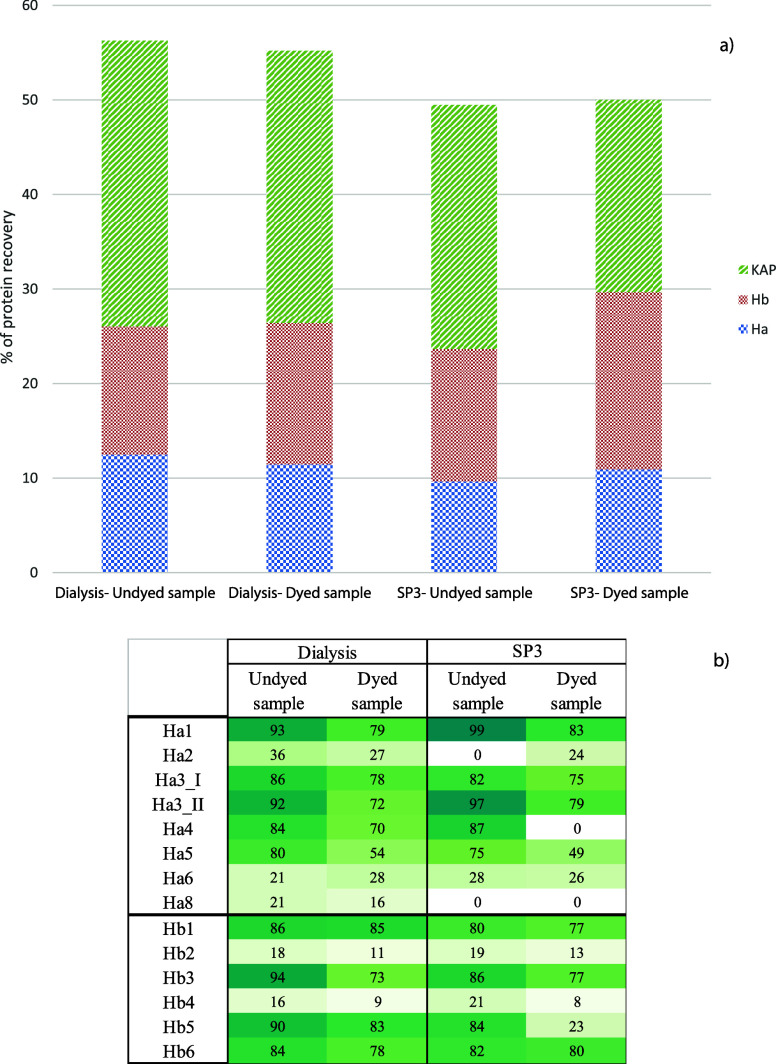
a) Percentage of protein recovery in the evaluation of the best protocol for the urea-Na_2_EDTA protocol with dialysis and SP3. b) Heatmap of coverage % for acid and basic keratins in the dialysis and SP3 experiments.

For the urea samples, dialysis seems to result in slightly better yields than SP3 based on the percentage of identified proteins (Figure 4a). Both protocols can extract different Ha and Hb proteins, but dialysis seems to be slightly better at preserving minor components, such as Ha8 in terms of sequence coverage (Figure 4b). Looking at the sequence coverage, the difference in the percentages have been evaluated following the percent difference (PD) comparison, calculated as indicated in the Supporting Information.

From this evaluation (Table S1), the comparison results between dialysis and SP3 are generally around 3% and 9% as difference values for the main components (i.e., Ha1 and Ha3_I), while the biggest differences can be appreciated for minor components (Ha6, Hb2, and Hb4), with values of 25%, 15% and 23%. Overall, two methods comparable from the point of view of protein coverage can be evaluated.

**Figure 5:**
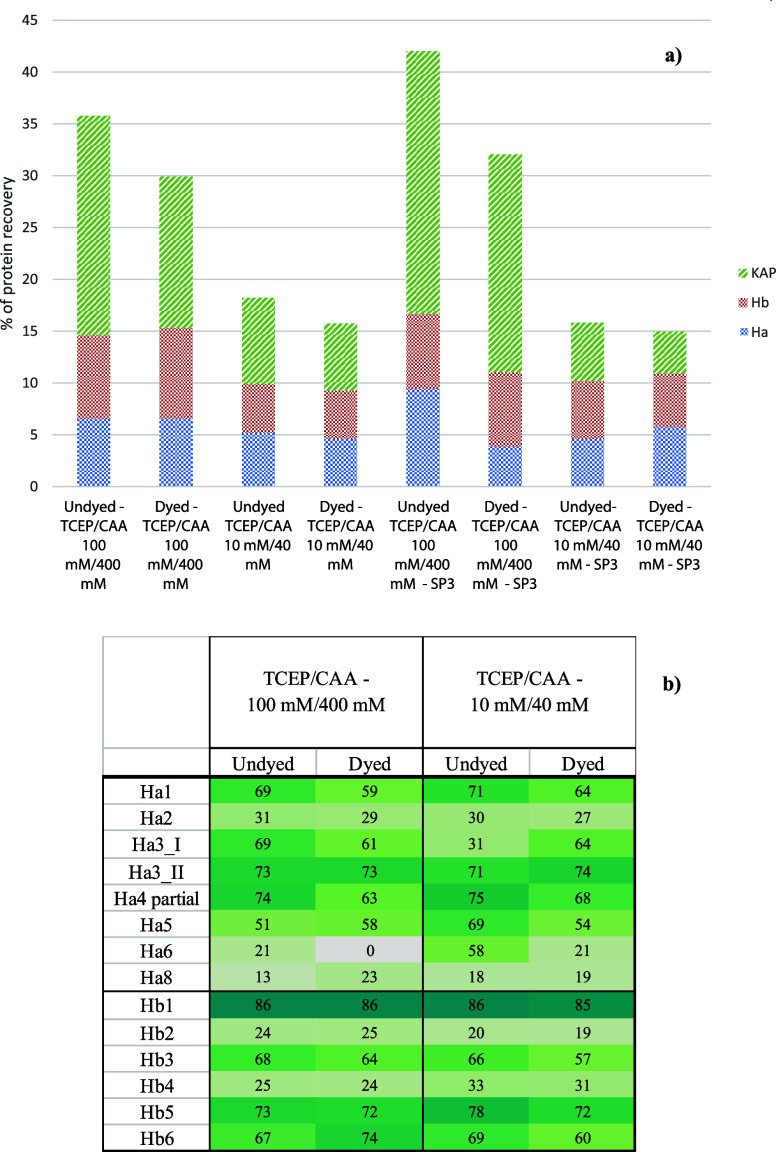
a) Percentage of protein recovery in the evaluation of the best protocol for different concentrations of TCEP/CAA, with and without SP3. b) Heatmap of coverage % for acid and basic keratins for the same experiments.

For the TCEP/CAA without SP3, comparing the higher and lower concentrations, the protein identification is higher as the number of protein groups identified for the 100 mM/400 mM solution (Figure 5a,b).

From the point of view of the percentage difference (table S2), in some cases the differences are significantly sharper, with this aspect more pronounced in the comparison of undyed samples. In fact, the highest absolute value is around 20% for Ha5, Ha8, and Hb4, while it exceeds 50% for Ha3_I and Ha6. In the rest of the cases, the values do not indicate marked differences in protein coverage.

Using SP3 slightly improves the recovery in protein composition, especially in the case of the undyed sample. However, the highest performance is driven by a higher TCEP/CAA concentration. However, it should be noted that if SP3 is done only on the soluble fraction, the results are almost zero, while they are completely different if performed on the whole samples (soluble fraction and insoluble fiber fraction together). This is in agreement with what reported in a previous study for the identification of fibers in textiles with heavy soil contamination, where it is hypothesized that with this procedure, the proteins are not really extracted from the matrix but more accessible for trypsin digestion.^16^

Once the concentration of TCEP/CAA was investigated, we evaluated different cleanup methods for dyes and proteins to assess whether the analysis of both components could lead to a loss of sensitivity or a decrease in the concentration of the two analytes, in effect compromising the possibility of analyzing the two components simultaneously.

**Figure 6:**
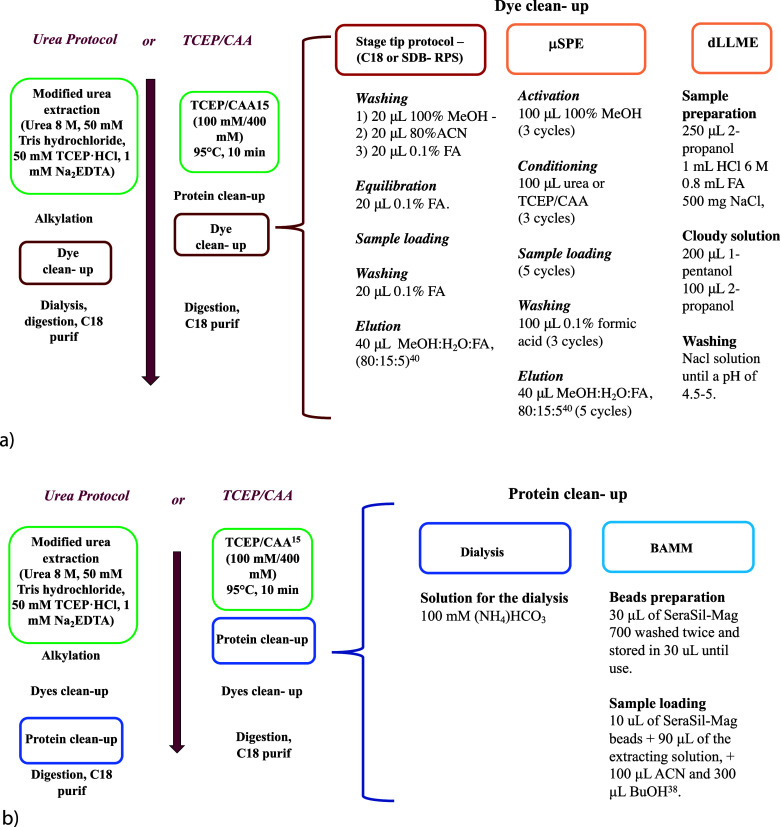
a) Schematic workflow for the different cleanup procedures for dyes; b) schematic workflow for the different cleanup procedures for proteins.

A scheme of the different cleanup procedures for dyes and proteins described afterward is presented in Figure 6a,b.

**Figure 7:**
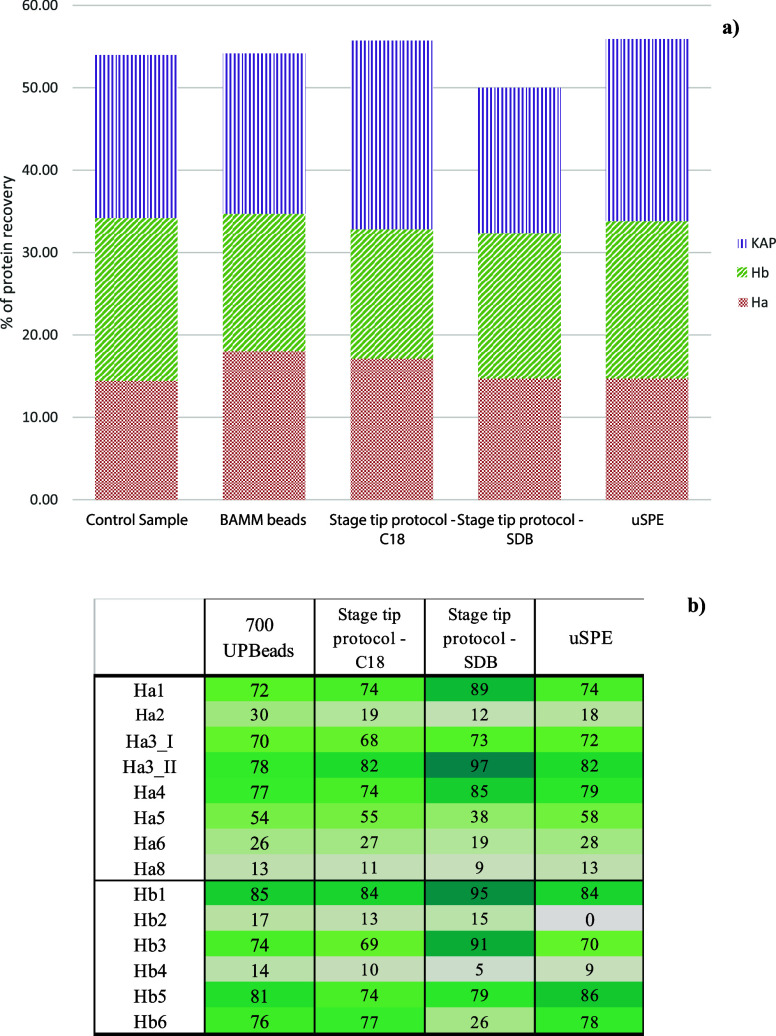
a) Percentage of protein recovery in the evaluation of several cleanups for dye recovery and isolation and how they affect the protein content. b) Heatmap of coverage % for acid and basic keratins for the same experiments.

Figure 7 shows an example of the extraction yield for the four cleanup methods described in the case of the urea extraction protocol. In this case, the four methods have been compared with a reference sample (i.e., a sample subjected to the protein extraction and characterization protocol without any dye cleanup steps).

As a general rule, after extraction in urea-Na_2_EDTA for 18 h, the solution was alkylated in iodoacetamide for 45 min and then subjected to clean up for dyes. The choice of alkylating first followed by the separation of dyes arouse from some tests performed on μ-SPE which demonstrated to be more effective in collecting dyes without removing proteins after the alkylation step and not before. This was then applied to the stage-tip protocol as well.

Once the stage-tip protocol or μ-SPE was performed, the remaining aqueous solution was subjected to dialysis and then trypsin digestion and C18 peptide purification or a stage-tip protocol for proteins. In the case of the BAMM beads, on the other hand, once the proteins were loaded onto the beads, digestion on beads was performed directly, while the solution was subjected to μ-SPE (or dLLME) to purify the dyes, which were then directly injected into the LC-MS system.

As described, the dye isolation performed through BAMM beads, applied for the first time on keratin proteins, and the stage-tip protocol with the SDB-RPS membrane not only seems to not affect the protein content (Figure 5b) but also provides the highest values for Ha (including minor components as Ha 2, 6, 8), while μ-SPE and the stage tip protocol with the C18 membrane provide the best results for Hb (including minor components) and KAP. The heatmap of protein coverage seems to confirm a general good coverage for most proteins, with a slight increase for the SDB-RPS sample. For the TCEP/CAA, since the best results were achieved with direct digestion on the fiber, the best protocol to collect the dyes appeared to be the μ-SPE performed before trypsin digestion.

**Figure 8:**
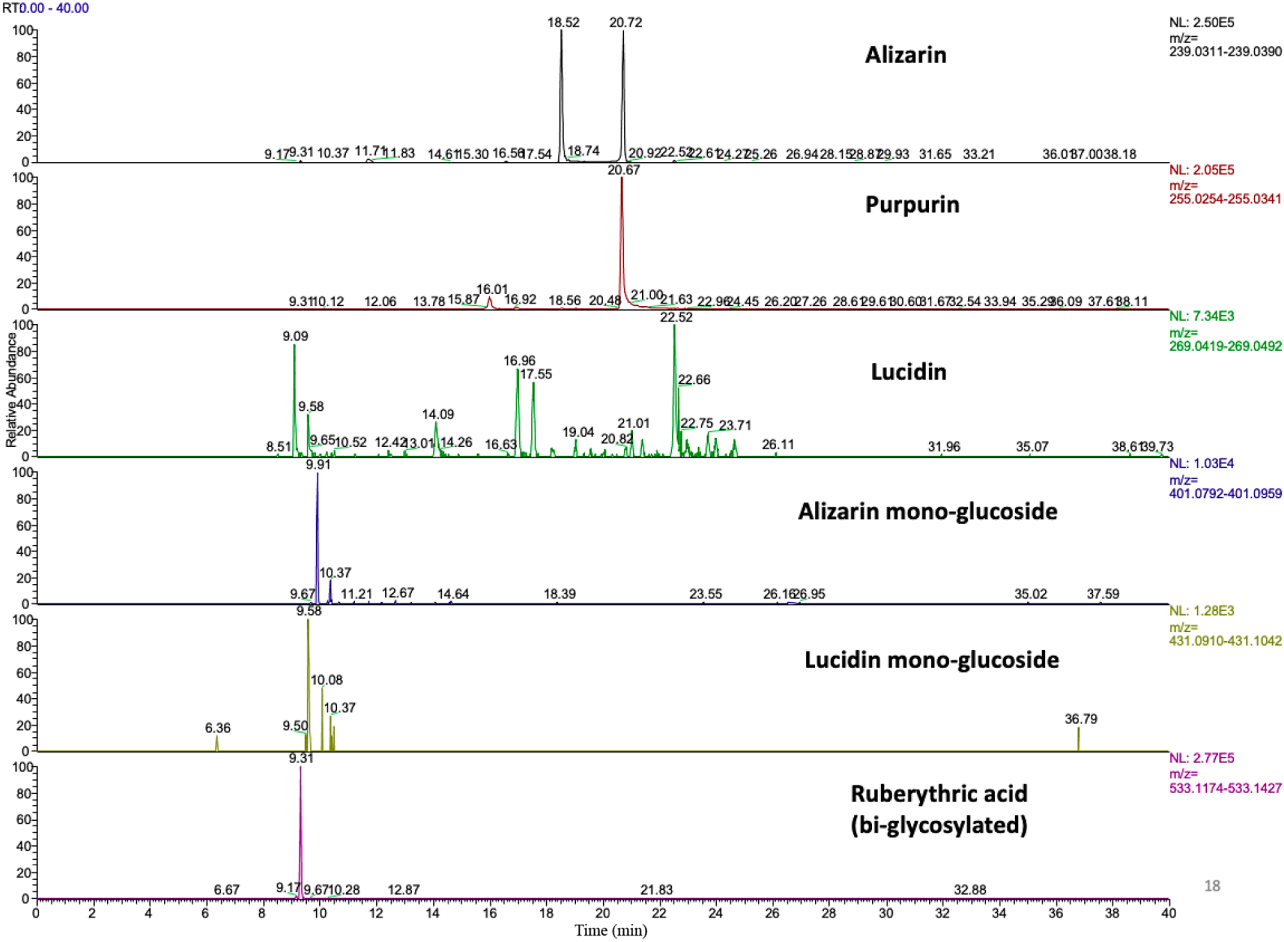
Extracted ion currents for the different dye molecules, in this case, from μ-SPE clean up.

For the dyes, basic environments have a greater impact on dye extraction, from a quality point of view;^21,33^ so, urea extraction leads to slightly better identification of dye components in the analyzed samples. Aglycone and glycosylated (mono- and billycosylated ones) were detected in both extracts from the two protocols (Figure 8), confirmed by MS/MS data.

**Figure 9:**
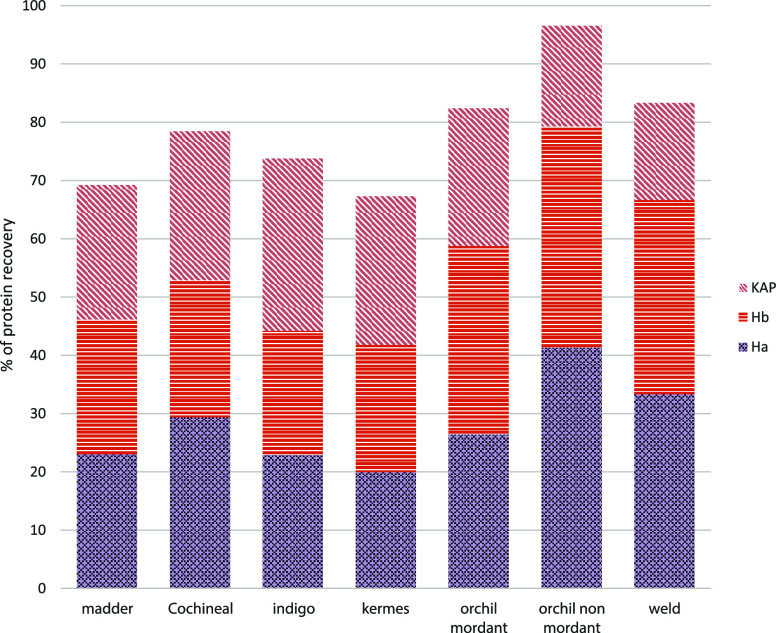
Percentage of protein recovery in the evaluation of urea extraction and dialysis for protein recovery from different dyed yarns.

Once the methods were developed, the urea method with BAMM/dialysis protocols for protein recovery and the stage tip protocol with SDB-RP membranes for dye recovery were then applied to other yarns dyed with different natural matrices, including cochineal and kermes (which share with madder the anthraquinone base structure), weld (flavonoid-based dyes), indigo (vat dye classes, indigoid structure), and orchil (direct dye class, phenoxazone structure). The results of extraction and characterization of proteins are provided in Figure 9.

Considering madder as a reference, it is possible to observe that in all cases the recoveries in terms of Ha, Hb, and KAP are comparable in %. Extraction from the nonmordant yarn of orchil however shows significantly higher recoveries for Ha and instead lower as KAPs, when compared with the other yarns, all of which have been subjected to mordant process. From this, it could be inferred that the mordanting process may have an effect on KAPs, which would merit further and specific investigation to be confirmed.

**Figure 10:**
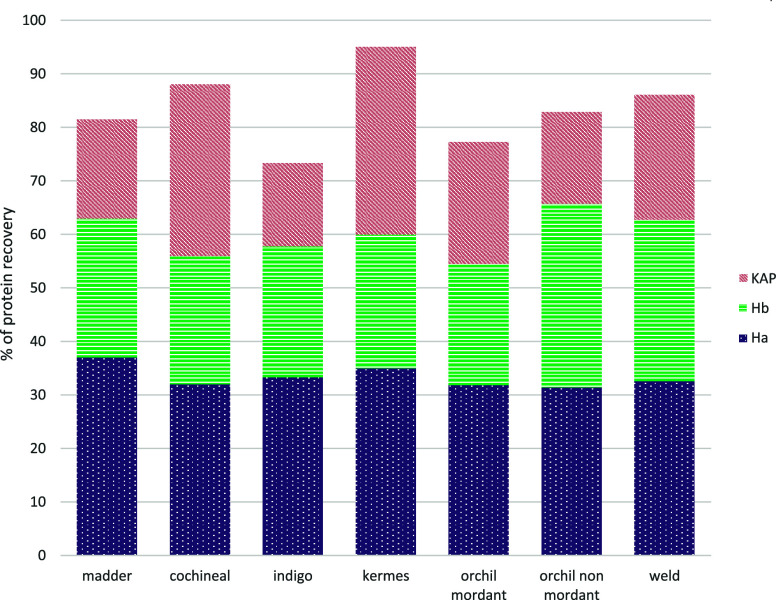
Percentage of protein recovery in the evaluation of urea extraction and the BAMM method for protein recovery from different dyed yarns.

However, when BAMM methodology is applied to urea extraction (Figure 10), the orchil nonmordant dyed sample does not show higher Ha recovery values, while still KAPs are lower. In this case, also for indigo-dyed yarns KAPs seem lower. Generally, again considering madder as a reference, it seems that, except for these two cases, the yields are comparable.

For the dyes, all the solutions appeared to be colored except the indigo sample after the urea extraction. For indigo, there is the possibility that indigo, as vat dyes, is not soluble in water solution but it requires a reduction, which brings it to its leucoform, not colored. Since the single-step solution contains TCEP, a reduction agent, it could extract indigo as leucoform, which is soluble in water instead.

**Figure 11:**
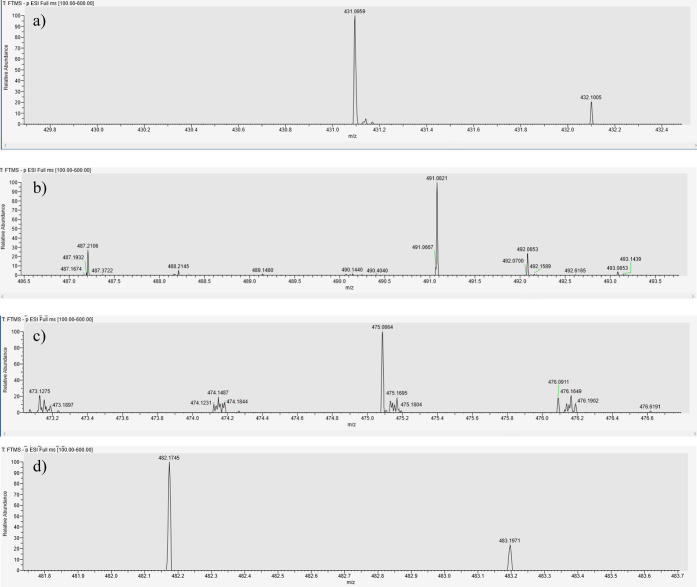
a) Mass spectrum of apigenin monoglycosylated (from weld samples). b) Mass spectrum of carminic acid (from cochineal samples). c) Mass spectrum of dcofk (o-glycosyl compound) from cochineal. d) Mass spectrum of β/γ amino-orcein from orchil.

Looking at the LC-HRMS analyses confirmed the identification of several compounds from the dye bath; in particular several glycosylated compounds were preserved (e.g., apigenin monoglycosylated from weld samples or dcofk, a glycosyl compound of flavokermesic acid, and carminic acid for cochineal dyed wool) demonstrating the effectiveness and the mild effect of this procedure (Figure 11a–d).

The signals for orcein dyes, even if present and detectable, were at a lower intensity, probably due to some difficulties in ionization of this molecules, which comprehend both hydroxyl and amino substituents. Indigo dyes or their leucoform have not been identified with certainty, which could suggest the need to develop conditions including ad hoc reductions for the extraction of indigo; nonetheless, it is worth to say that indigo is a molecule easily recognizable even with spectroscopic techniques unlike other dyes.

## Conclusions and Future Perspective

The aim of the work was to develop a protocol that would ensure that proteins and dyes could be analyzed from textile specimens by a single extraction step and identify an integrated workflow. Both the urea and TCEP/CAA protocols are effective in extracting both proteins and dye in a single step. The methodology proved to be effective not only on dyed yarns but also on a large variety of keratin-dyed materials. In particular, it demonstrated the ability to extract direct dyes and mordant dyes. This is of particular interest since it would mean the procedure is effective for most types of dyes. Using two different protocols gives us the chance to easily adapt this integrated approach to the state of conservation of the artifacts. Several clean up procedures also are promising: μ-SPE and the stage-tip protocol with the SDB-RPS polymer for dye analyses and the paramagnetic bead-assisted cleanup for protein, which dramatically reduce sample preparation time and improve the total number of protein groups extracted compared to a control sample.

As mentioned in the Introduction, these protocols represent the first step in a larger project, PARCA, founded by the European commission, with the aim of having a new insight into the characterization of charred and degraded textiles. The protocols will be applied first to thermally aged textiles to evaluate the effectiveness of the methodology when thermal aging-like modifications occur. In the last part of the project, it is anticipated to study archeological samples from the Mediterranean Sea (especially Pompeii and Vesuvian area and Greek area, from Eubea island, Athen’s area and in the southern part of Aetolia-Acarnania).

## Supporting Information Available

Comparison of coverage % for acid and basic keratins in the dialysis and SP3 experiments for the rea extraction protocol (Table S1); comparison of coverage % for acid and basic keratins in the TCEP/CAA extraction protocol, with and without SP3 (Table S2) (PDF)
