# Supplementary material for: Development of a Combined Protein and Dye Extraction Approach for the Analysis of Keratin-Based Textiles
Source: J Proteome Res. 2024 Aug 8;23(9):3890–903. doi: 10.1021/acs.jproteome.4c00253 (PMC11385382; doi:10.1021/acs.jproteome.4c00253)
Supplement: Supplementary file 1 — pr4c00253_si_001.pdf [file pr4c00253_si_001.pdf]

Supporting Information

Development of a Combined Protein and Dye Extraction Approach for the Analysis of keratin-Based Textiles

Ilaria Serafini[1,2,3], Gabriele Favero[3], Roberta Curini[1], Gwénaëlle M. Kavich[2], Timothy P. Cleland[2]*

1-Dept of Chemistry, Sapienza University of Rome, Piazzale Aldo Moro 5, 00185 Rome, Italy

2-Museum Conservation Institute, Smithsonian Institution, 4210 Silver Hill Rd, Suitland, MD, 20746, United States

3-Dept of Environmental Biology, Sapienza University of Rome, Piazzale Aldo Moro 5, 00185 Rome, Italy

KEYWORDS

Keratins; keratins associated proteins; dyes; archaeological textiles; paramagnetic beads

****

Table of content – supporting information

**Supporting element**	**Caption**
Table S1	Comparison of coverage % for acid and basic keratins in the dialysis and SP3 experiments for urea extraction protocol.
Table S2	Comparison of coverage % for acid and basic keratins in TCEP/CAA extraction protocol, with and without SP3

The percent difference (PD) comparison was calculated following the formula:

$$PD = \left(\frac{(n2 - n1)}{n2} \right) x\ 100$$

Where n1 = the smaller of the two numbers being compared and n2 = the larger of the two numbers being compared.

The following tables, S1 and S2, represent the comparison of coverage %.

	**PD – undyed (%)**	**PD- dyed (%)**
Ha1	6	5
Ha2	na	11
Ha3_I	5	4
Ha3_II	5	9
Ha4	3	na
Ha5	6	9
Ha6	25	7
Ha8	na	na
Hb1	7	9
Hb2	5	15
Hb3	9	5
Hb4	24	11
Hb5	7	72
Hb6	2	3

Table S1- Comparison of coverage % for acid and basic keratins in the dialysis and SP3 experiments for urea extraction protocol.

	PD- undyed (%)	PD – dyed (%)
Ha1	3	8
Ha2	3	7
Ha3_I	55	5
Ha3_II	3	1
Ha4	1	7
Ha5	26	7
Ha6	64	na
Ha8	28	17

Hb1	0	1
Hb2	17	24
Hb3	3	11
Hb4	24	23
Hb5	6	0
Hb6	3	19

Table S2- Comparison of coverage % for acid and basic keratins in TCEP/CAA extraction protocol, with and without SP3.
